# Detailed visual assessment of striatal dopaminergic depletion in patients with idiopathic normal pressure hydrocephalus: unremarkable or not?

**DOI:** 10.1186/s12883-020-01861-7

**Published:** 2020-07-11

**Authors:** Jeong-Yoon Lee, Soo Bin Park, Mina Lee, Hyunjin Ju, Kayeong Im, Kyum-Yil Kwon

**Affiliations:** 1 Department of Neurology, Soonchunhyang University Seoul Hospital, Soonchunhyang University School of Medicine, 59 Daesagwan-ro, Yongsan-gu, Seoul, 04401 Republic of Korea; 2 Department of Nuclear Medicine, Soonchunhyang University Seoul Hospital, Soonchunhyang University School of Medicine, Seoul, Republic of Korea

**Keywords:** Dopamine transporter imaging, FP-CIT PET, Normal pressure hydrocephalus, Striatal dopamine

## Abstract

**Background:**

Dopamine transporter (DAT) imaging may enable clinicians to discriminate idiopathic normal pressure hydrocephalus (iNPH) from other parkinsonian disorders. However, a specific pattern of dopaminergic loss in DAT imaging of iNPH patients remains to be further elucidated.

**Methods:**

In this preliminary study, 11 patients with iNPH in our hospital between March 2017 and February 2019 were finally enrolled. A diagnosis of iNPH was made according to the two established criteria. For visual analysis of DAT imaging, a striatum was divided into five domains. A semi-quantitative visual assessment was performed with a consensus between a nuclear medicine specialist and an experienced neurologist who were blinded to the clinical diagnosis.

**Results:**

Striatal dopaminergic deficits were abnormal in 90.9% (10/11) of patients with iNPH. The degree of dopaminergic reduction was mild and heterogeneous. However, a tendency of preferential striatal DAT loss in the caudate nucleus (90.9%, 10/11) than in the putamen (72.7%, 8/11) was observed, whereas ventral portion (9.1%, 1/11) was relatively preserved.

**Conclusion:**

Striatal dopaminergic depletion might be mild and heterogeneous in patients with iNPH. These dopaminergic deficits were more common in the caudate nucleus than in the putamen, suggesting a pattern different from other degenerative parkinsonian disorders.

## Background

Various degrees of parkinsonism and the cardinal symptom triad of gait disturbance, cognitive decline, and urinary incontinence are commonly observed in patients with idiopathic normal pressure hydrocephalus (iNPH). These symptoms of iNPH can be relieved by surgical interventions. In this regard, discriminating iNPH from its mimics such as Parkinson’s disease (PD) and Parkinson-plus syndrome is tremendously challenging in clinical settings [1].

Among several diagnostic imaging modalities, dopamine transporter (DAT) imaging is useful for the diagnosis of degenerative parkinsonian disorders. According to a previous study, PD and Parkinson-plus syndrome showed different subregional patterns of striatal DAT loss in ^18^F-N-(3-fluoropropyl)-2β-carboxymethoxy-3β-(4-iodophenyl) nortropane (FP-CIT) positron emission tomography (PET) images. Images of ^18^F-FP-CIT PET in patients with PD, progressive supranuclear palsy and multiple system atrophy showed different preferential DAT loss in dorsal posterior putamen, caudate nucleus and putamen, and ventral and dorsal posterior putamen, respectively [2, 3]. Although Ouchi et al. [4] reported that presynaptic dopaminergic depletion was not observed in patients with iNPH, not every iNPH patients showed normal DAT imaging in recent studies. According to reports of Broggi et al. [5] and Allali et al. [6], abnormal DAT losses were seen in 46.5 and 31% of iNPH patients with parkinsonism, respectively. However, a specific pattern of dopaminergic loss in DAT imaging of iNPH patients has not been reported yet.

Thus, the aim of this preliminary study was to identify a specific pattern of DAT imaging in patients with iNPH using ^18^F-FP-CIT PET by visual assessment. This study hypothesizes that the subregional pattern of dopaminergic loss in iNPH might be discernible from those in patients with its mimics described above. Results of this study could contribute to the understanding of the exact pathophysiology of iNPH which has not been fully figured out yet.

## Methods

### Subjects

Medical records and brain imaging results of 20 patients diagnosed with iNPH who had come to the movement disorder clinic in our hospital between March 2017 and February 2019 were retrospectively reviewed. A diagnosis of iNPH was based on clinical history and brain magnetic resonance imaging (MRI) at 3 T features that fulfilled requirements of both “probable iNPH” from the iNPH consensus guideline criteria [7] and “possible iNPH with MRI support” from the Japanese iNPH guideline criteria [8]. All of the followings were required for the diagnosis of iNPH: gait disturbance plus at least one of the other symptoms including cognitive impairment, urinary incontinence, or both; ventricular enlargement (Evans’ index > 0.3); narrowing of the sulci and subarachnoid spaces over the high convexity/midline surface on brain MRI; cerebrospinal fluid opening pressure lower than 200 mmH_2_O measured with a lumbar puncture or a comparable procedure; and the absence of severe medical illness or preceding diseases that could cause secondary NPH. To rule out neurodegenerative parkinsonian disorders, any patient with a potential diagnosis of PD or Parkinson plus syndrome showing typical clinical symptoms (asymmetrical parkinsonism, supranuclear gaze palsy, or prominent orthostatic hypotension) and/or neuroradiological features (prominent brainstem or cerebellar atrophy, or asymmetric cortical atrophy) was excluded. Also, patients with the evidence of cerebrovascular or other structural cerebral diseases which could interfere with the interpretation of ^18^F-FP-CIT PET findings were excluded in the evaluation of brain 3 T MRI, including fluid attenuated inversion recovery image. Finally, a total of 11 patients with iNPH were enrolled in the study. In every patient, the severity of cognitive impairment, gait disturbance, and urinary symptom were assessed with the validated iNPH grading scale [9]. The data set of the present study was anonymized and de-identified.

### Techniques for positron emission tomography

All enrolled subjects underwent ^18^F-FP-CIT PET using a PET/computed tomography (CT) scanner (Biograph mCT, Siemens Healthcare, Erlangen, Germany) with an in-plane spatial resolution of 2.0-mm full width at half maximum from the center of the field of view. Prior to PET/CT scanning, all subjects had discontinued medications that could affect the affinity of radioligands [10]. After injecting 185 MBq of ^18^F-FP-CIT intravenously, image acquisition was performed 2 h later. CT images were acquired on a 128-slice helical CT with 120 KV and 100 mA adjusted to body weights using an automatic exposure control (slice thickness, 3.0 mm). Emission scans were then obtained for 10 min. CT-based attenuation corrected PET images were then reconstructed using an iterative algorithm with point spread function and time-of-flight (5 iterations and 21 subsets). Maximal intensity projection (MIP) image was made at an angle of 12 degrees.

### Visual assessments

In every patient, both 2D- and 3D-images of ^18^F-FP-CIT PET were visually assessed by the consensus of a nuclear medicine specialist (SBP) and an experienced neurologist (JYL). Both physicians were totally blinded to the clinical information of subjects. A striatum was divided into five domains: anterior caudate nucleus (AC); posterior caudate nucleus (PC); anterior putamen (AP); posterior putamen (PP); and ventral striatum (VS) [2]. MIP, axial, coronal, and sagittal PET images were simultaneously investigated using an image viewing software Syngo.via (Siemens Healthcare, Erlangen, Germany). The color of PET image was rainbow. Radioactivity was manually adjusted with background brain activity in blue color. Considering the age- and gender-related effects on DAT availability, we utilized normal DAT images of age- and gender-matched controls in our hospital (Additional file 1). FP-CIT bindings in each striatal subregion were graded by visual comparison to a normal striatal subregion as reference (Additional file 2): 0, no significant reduction; 1, mild reduction; 2, moderate reduction; and 3, severe reduction or sparse radioactivity [11]. A semi-quantitative visual analysis (Additional file 3) was then dichotomized into normal (0) and abnormal (1, 2, 3) in each subregion of the striatum for the following reasons. Firstly, most of abnormal regions were graded as mild reduction. Secondly, detailed data were too heterogeneous to obtain any uniform or meaningful result. Thirdly, the number of enrolled patients was relatively small to be analyzed in detail.

## Results

### 2D- and 3D-images of ^18^F-FP-CIT PET in patients with idiopathic normal pressure hydrocephalus

**Fig. 1:**
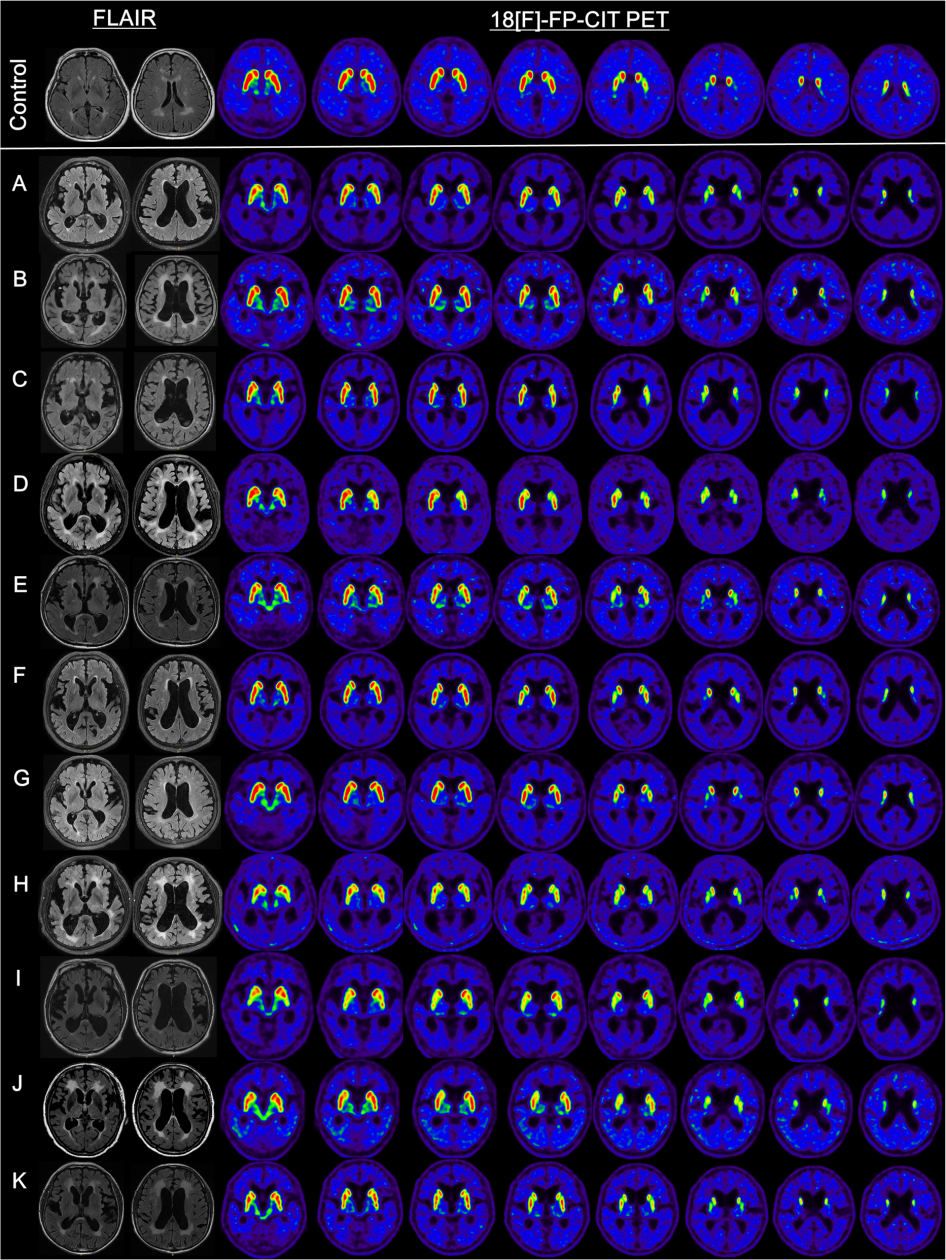
Fluid-attenuated inversion recovery and transaxial images of 18F-FP-CIT PET of the control (top line) and 11 subjects with idiopathic normal pressure hydrocephalus (Case A-K)

**Fig. 2:**
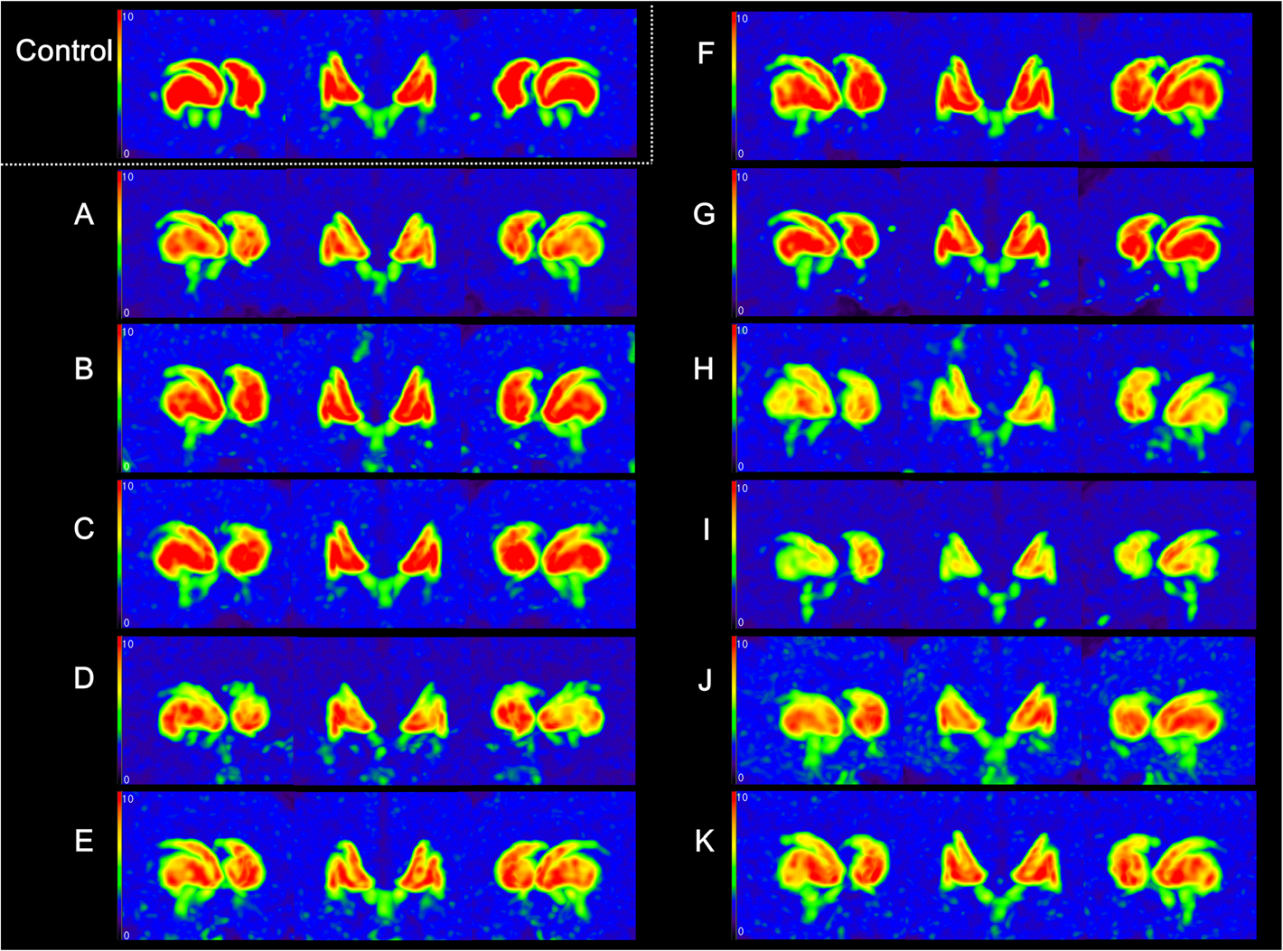
Maximal intensity projection images of 18F-FP-CIT PET of the control and every subject with idiopathic normal pressure hydrocephalus

Clinical characteristics and severities of iNPH symptoms are described in Table 1. Age of subjects ranged from 74 to 90 years. Duration of the disease at the time of obtaining PET images ranged from 6 months to 5 years. All subjects (except case H) had no history of cerebrovascular diseases. For case H, a small 2 mm-sized focal intracerebral hemorrhage was present in the left thalamus on brain MRI which was thought to be unrelated to the basal ganglia pathology. Every enrolled patient had all iNPH symptoms including gait disturbance, memory disturbance, and urinary symptom. Fluid-attenuated inversion recovery (FLAIR) and transaxial images of ^18^F-FP-CIT PET are depicted in Fig. 1. FLAIR images of all patients with iNPH showed various degrees of white matter hyperintensity and ventriculomegaly with Evans’ index of more than 0.3, while no significant structural lesion in the basal ganglia was noted. MIP images of ^18^F-FP-CIT PET are displayed in Fig. 2. Striatal DAT loss revealed significantly heterogenous patterns in subjects. Out of 11 patients, 8 patients showed abnormal ^18^F-FP-CIT binding in both striatum. In case A and case C, unilateral abnormal results were found in the left stratum and the right striatum, respectively. Normal ^18^F-FP-CIT PET results in both striatum were observed only in case G. Regarding the caudate nucleus, 10 patients showed abnormal ^18^F-FP-CIT PET results. Among these 10 patients, PC was more affected than AC in 7 patients, while PC and AC were affected similarly in 3 patients. As for the putamen, abnormal PET results were found in 8 patients. Among these 8 patients, PP was more affected than AP in 7 patients, while PP and AP were similarly affected in 1 patient. A reduction of ^18^F-FP-CIT binding to VS was observed only in Case I. Severe DAT loss (visually graded as 3) was found in PC (22.7%, 5 out of 22 striatum) and PP (9.1%, 2 out of 22 striatum). Asymmetrical DAT loss was observed in 7 (63.6%) patients.

**Table 1:** Clinical characteristics of patients with idiopathic normal pressure hydrocephalus

	A	B	C	D	E	F	G	H	I	J	K
Disease duration (yr)	1	2	2	3	0.5	1	1	0.5	5	5	2
Education (yr)	16	6	4	0	12	12	9	9	6	12	NA
Comorbidity	HTN	DM	Tibial fracture	HTN/RA	DM/HTN/Angina	HTN	DM/HTN/ angina	Focal ICH	DM/HTN	HTN/CKD	Gastric cancer
NPH grading scale											
Gait (0–3)	2	3	2	3	2	2	2	2	3	3	2
Cognition (0–3)	2	3	1	3	2	2	1	1	2	3	2
Urinary (0–3)	1	2	2	3	2	2	1	1	2	3	1

Abbreviations: *NA* not applicable, *HTN* hypertension, *DM* diabetes mellitus, *RA* rheumatoid arthritis, *ICH* intracerebral hemorrhage, *CKD* chronic kidney disease

### Abnormal proportion of ^18^F-FP-CIT PET in patients with idiopathic normal pressure hydrocephalus

**Fig. 3:**
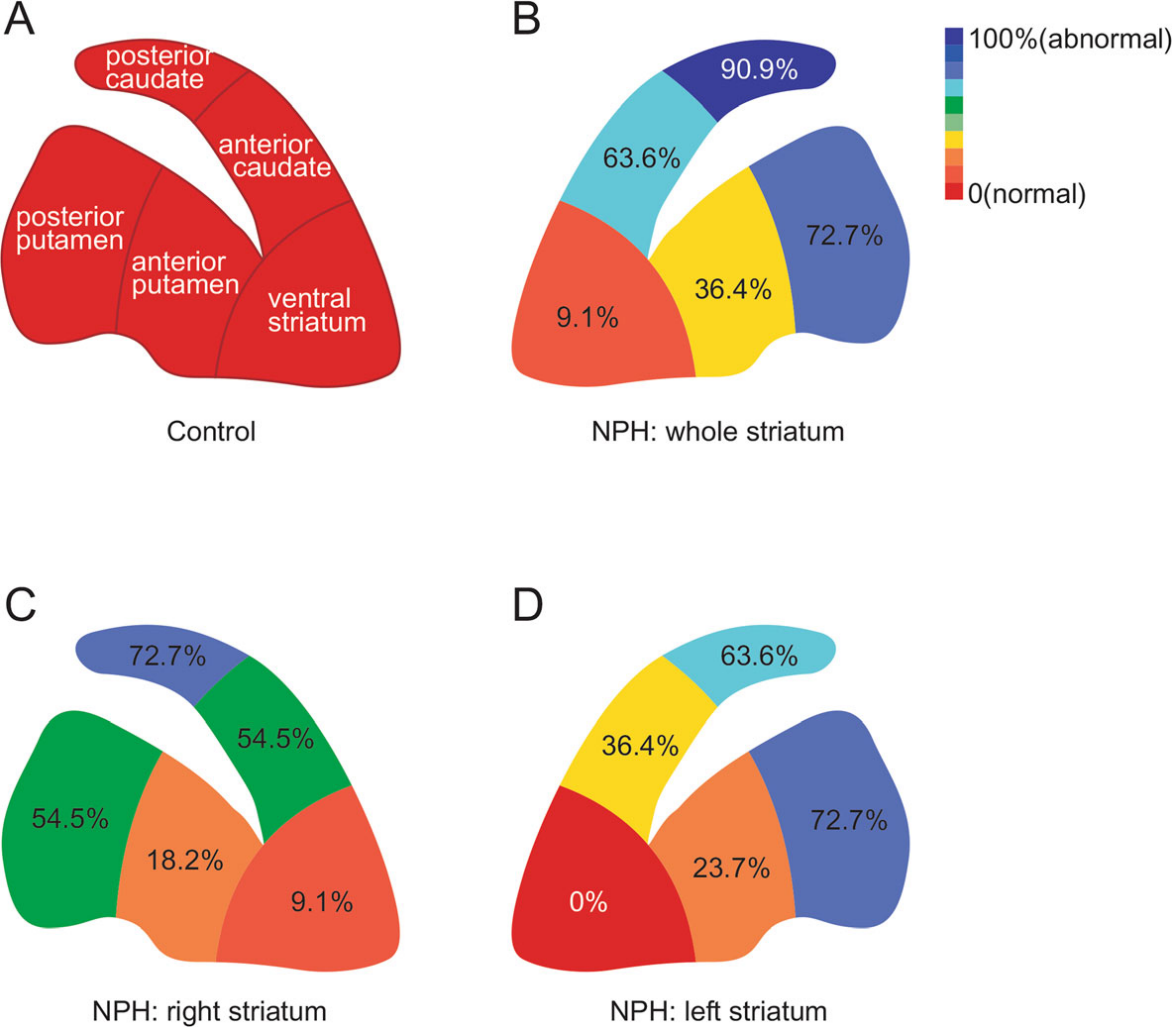
A proportion of idiopathic normal pressure hydrocephalus patients showing abnormal 18F-FP-CIT PET results in each striatal subregion

A proportion of iNPH patients showing abnormal ^18^F-FP-CIT PET results in each striatal subregion is depicted in Fig. 3. PC and PP were abnormal in 90.9 and 72.7% of subjects, respectively, whereas VS was abnormal in only 9.1% of subjects (Fig. 3b). In the right striatum, PC, PP, AC, and VS were abnormal in 72.7, 54.5, 54.5, and 9.1% of subjects, respectively (Fig. 3c). In the left striatum, PC, PP, AC, and VS were abnormal in 63.6, 72.7, 36.4, and 0% of subjects, respectively (Fig. 3d).

## Discussion

In this study, results of ^18^F-FP-CIT PET were abnormal in 90.9% (10/11) of enrolled iNPH patients, showing a remarkably heterogenous pattern. With regard to striatal subregions, a tendency of preferential striatal DAT loss in the caudate nucleus than that in the putamen was observed, whereas VS was relatively preserved. Additionally, a pattern of rostrocaudal gradient was found in the caudate nucleus and putamen, with the caudate nucleus being affected more than the putamen. These characteristics of presynaptic dopaminergic loss in iNPH are quite discernible from those in PD which tends to affect dorsal posterior putamen with relatively preserved caudate nucleus [2]. To the best of our knowledge, this is the first study to report a specific pattern of DAT loss in iNPH patients using ^18^F-FP-CIT PET.

There have been a few reports investigating presynaptic dopaminergic deficit in hydrocephalic patients using different radiotracers. In a previous study using [^11^C]2-b-carbomethoxy-3b-(4-fluorophenyl) tropane, presynaptic dopaminergic loss was not found in iNPH patients [4]. However, in that study, all enrolled patients had only mild severity of gait disturbance, which could be related to the relatively preserved dopaminergic function in the substantia nigra of the midbrain [12]. In a study of Broggi et al. [6], 14 (46.5%) out of 30 iNPH patients with parkinsonism showed abnormal results in [^123^I]FP-CIT single photon emission computed tomography (SPECT) imaging, although subregional patterns of striatal dopaminergic loss were not described in that study. In a report of Allali et al. [5], 46.2 and 31.8% of iNPH patients showed abnormal [^123^I]FP-CIT SPECT by visual rating scale and semi-quantitative analysis, respectively. Although normal [^123^I]FP-CIT SPECT results on the basis of visual rating scale were not associated with a diagnosis of iNPH, normal results in the whole striatum by semi-quantitative analysis were associated with a diagnosis of iNPH. However, with respect to striatal subregions, normal caudate nucleus was not related to iNPH, while normal putamen was equivocal. In a case of obstructive hydrocephalus with shunt malfunction, abnormal 6-[^18^F] fluorodopa PET scan was found in the caudate nucleus and putamen with a relatively low caudate/posterior putamen ratio [13]. These outcomes could support results of our study which revealed a high tendency of dopaminergic loss in the caudate nucleus and putamen, particularly in the PC of patients with iNPH.

Several hypotheses can be postulated for the presynaptic dopaminergic loss in iNPH patients. A few articles have demonstrated a morphological alteration of the midbrain in iNPH patients [4, 12]. As a consequence, an injury of the substantia nigra and/or striatum by abnormal pulsatile CSF flow can induce a dysfunction of the nigrostriatal pathway [7]. Characteristics of the basal ganglia include high metabolic rate, distinctive microvasculature, and autoregulation. Other than PD and Parkinson-plus syndrome, diseases having structural lesions in the basal ganglia such as vascular parkinsonism and Fahr disease show presynaptic dopaminergic loss on DAT imaging and reduced cerebral blood flow in the basal ganglia on perfusion imaging [14, 15]. Likewise, regional cerebral blood flow is remarkably reduced in the caudate nucleus, putamen, and thalamus in iNPH patients [16]. In addition, direct compression of the basal ganglia by ventricular enlargement could contribute to a dysfunction as well as a reduction in the size of the basal ganglia, especially the caudate nucleus. Moreover, a decrease of glucose metabolism which shows high accordance rate with perfusion SPECT has been observed in the basal ganglia of a patient with iNPH [17, 18]. These findings suggest that the dysfunction or degeneration of basal ganglia could attribute to the pathophysiology of iNPH, in accordance with a neuropathological study [19]. In our study, every enrolled iNPH patient had all of the cardinal symptom triad, meaning that patients at early stages of iNPH were not included. Although relationship between the severity of the clinical symptoms and striatal dopaminergic depletion has not been elucidated, we suppose that it might have affected the results of our study.

Our preliminary study has several strengths and shortcomings. Firstly, we used ^18^F-FP-CIT PET known to be more sensitive than other DAT imaging tools including [^123^I]FP-CIT SPECT [18, 20]. To the best of our knowledge, a study investigating iNPH patients with ^18^F-FP-CIT PET has not been reported yet. Secondly, we used both the iNPH consensus guideline criteria and the Japanese iNPH guideline criteria for the exact diagnosis of iNPH in every patient [7, 8]. Thirdly, all patients underwent both MRI and DAT imaging. As for shortcomings of this study, firstly, the number of subjects was too small and we did not assess the detailed parkinsonian motor symptoms using UPDRS part III. Accordingly, we could not investigate the association between DAT loss and the severity of parkinsonian motor symptoms in iNPH. Secondly, we used semi-quantitative visual assessment known to be less sensitive than automatic quantitative analysis using region of interest. Thirdly, since none of our subjects underwent a postmortem examination, a possibility of the coexistence of iNPH and other parkinsonian neurodegenerative diseases might exist [21, 22].

## Conclusion

In conclusion, mild depletion of striatal dopamine with a rostrocaudal gradient was found in patients with iNPH. Moreover, dopaminergic deficit might be more common in the caudate nucleus than in the putamen. This is a distinctive pattern, unlike PD or Parkinson-plus syndrome. Results of this study could be used as a cornerstone to highlight the clinical significance of DAT imaging for the diagnosis and understanding of the exact pathophysiology of iNPH. Further studies are needed not only to confirm results of this preliminary study, but also to find out the associations between striatal dopaminergic depletion and parkinsonian motor symptoms in patients with iNPH.

## Supplementary information

**Additional file 1.** Maximal intensity projection images of 18F-FP-CIT PET of age- and gender-matched controls.

**Additional file 2.** A representative example of semi-quantitative visual assessment of a patient with idiopathic normal pressure hydrocephalus (Case I).

**Additional file 3.** 18F-FP-CIT PET consensus read results of patients with idiopathic normal pressure hydrocephalus.

## Data Availability

The datasets used and/or analyzed during the study are available from the corresponding author on reasonable request.

## Abbreviations

iNPH: Idiopathic normal pressure hydrocephalus
PD: Parkinson’s disease
DAT: Dopamine transporter
FP-CIT: ^18^F-N-(3-fluoropropyl)-2β-carboxymethoxy-3β-(4-iodophenyl) nortropane
PET: Positron emission tomography
MRI: Magnetic resonance imaging
CT: Computed tomography
MIP: Aximal intensity projection
AC: Anterior caudate
PC: Posterior caudate
AP: Anterior putamen
PP: Posterior putamen
VP: Ventral striatum
FLAIR: Fluid-attenuated inversion recovery
SPECT: Single photon emission computed tomography
