# Supplementary figures and images for: Detailed visual assessment of striatal dopaminergic depletion in patients with idiopathic normal pressure hydrocephalus: unremarkable or not?
Source: BMC Neurol. 2020 Jul 11;20:277. doi: 10.1186/s12883-020-01861-7 (PMC7353728; doi:10.1186/s12883-020-01861-7)

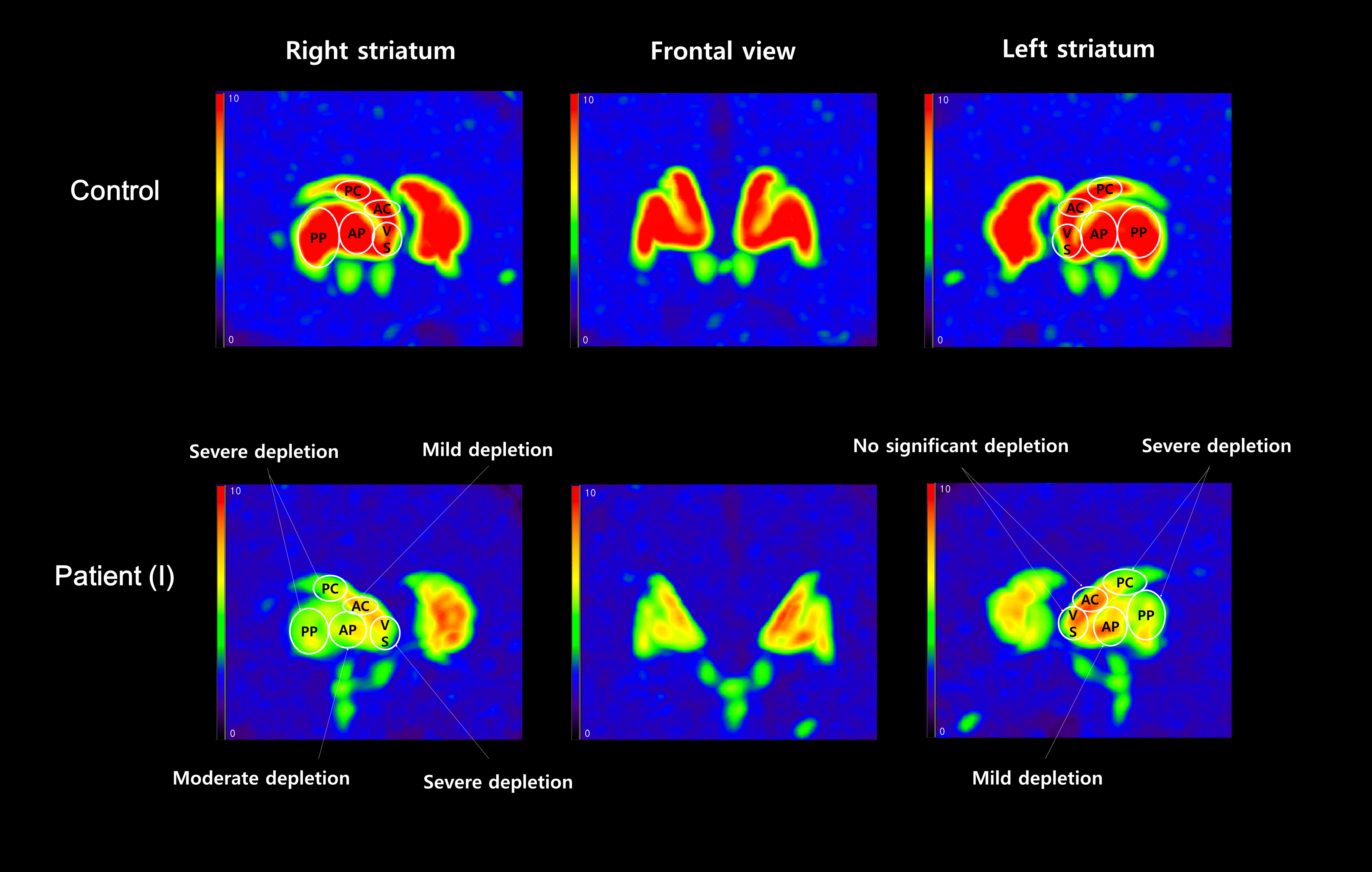

Supplement: Supplementary file 2 — Additional file 2. A representative example of semi-quantitative visual assessment of a patient with idiopathic normal pressure hydrocephalus (Case I). [file 12883_2020_1861_MOESM2_ESM.tif]
