# Supplementary material for: Detailed visual assessment of striatal dopaminergic depletion in patients with idiopathic normal pressure hydrocephalus: unremarkable or not?
Source: BMC Neurol. 2020 Jul 11;20:277. doi: 10.1186/s12883-020-01861-7 (PMC7353728; doi:10.1186/s12883-020-01861-7)
Supplement: Supplementary file 3 — Additional file 3. 18F-FP-CIT PET consensus read results of patients with idiopathic normal pressure hydrocephalus. [file 12883_2020_1861_MOESM3_ESM.docx]

**Supplementary Table 1. [^18^F]-FP-CIT PET consensus read results of patients with idiopathic normal pressure hydrocephalus**

| Case | Right striatum | | | | |  | Left striatum | | | | |
| --- | --- | --- | --- | --- | --- | --- | --- | --- | --- | --- | --- |
|  | AC | PC | AP | PP | VS |  | AC | PC | AP | PP | VS |
| A | 0 | 0 | 0 | 0 | 0 |  | 1 | 1 | 0 | 1 | 0 |
| B | 0 | 1 | 0 | 1 | 0 |  | 0 | 1 | 0 | 1 | 0 |
| C | 1 | 1 | 0 | 0 | 0 |  | 0 | 0 | 0 | 0 | 0 |
| D | 2 | 3 | 0 | 0 | 0 |  | 2 | 3 | 2 | 2 | 0 |
| E | 0 | 0 | 0 | 1 | 0 |  | 0 | 1 | 0 | 1 | 0 |
| F | 0 | 1 | 0 | 1 | 0 |  | 0 | 1 | 0 | 1 | 0 |
| G | 0 | 0 | 0 | 0 | 0 |  | 0 | 0 | 0 | 0 | 0 |
| H | 2 | 2 | 1 | 2 | 0 |  | 2 | 2 | 2 | 2 | 0 |
| I | 1 | 3 | 2 | 3 | 3 |  | 0 | 3 | 1 | 3 | 0 |
| J | 1 | 3 | 0 | 0 | 0 |  | 0 | 2 | 0 | 0 | 0 |
| K | 1 | 2 | 0 | 1 | 0 |  | 1 | 2 | 0 | 1 | 0 |

Abbreviations: AC, anterior caudate nucleus; PC, posterior caudate nucleus; AP, anterior putamen; PP, posterior putamen; VS, ventral striatum.

Visual rating scale was classified as normal or no reduction (0), mild reduction (1), moderate reduction (2), and severe reduction (3).
